# Risk factors for and management of anterior chamber intravitreal dexamethasone implant migration

**DOI:** 10.1186/s12886-019-1122-1

**Published:** 2019-05-28

**Authors:** D. Röck, K. U. Bartz-Schmidt, T. Röck

**Affiliations:** Centre for Ophthalmology, University of Tübingen, Elfriede-Aulhorn-Straße 7, D-72076 Tübingen, Germany

**Keywords:** Anterior chamber migration, Corneal edema, Dexamethasone implant, Explantation, Ozurdex®, Vitrectomy

## Abstract

**Background:**

This study aimed to investigate the incidence of and risk factors for the anterior chamber migration of an intravitreal dexamethasone implant (Ozurdex®).

**Methods:**

A retrospective review of 640 consecutive intravitreal dexamethasone implant injections was conducted from February 2011 through February 2018 at the University Eye Hospital in Tübingen, Germany. Those patients who experienced anterior chamber dexamethasone implant migrations were identified, as well as the reasons for the anterior chamber migration. The surgical histories were obtained and comprehensive ophthalmic examinations were conducted for all of the eyes. Cross-tabulations, chi-squared tests, and Fisher’s exact tests were used to assess the influences of different factors on the anterior chamber implant migrations.

**Results:**

Overall, 4 eyes of four patients (0.63%) showed anterior chamber implant migrations. All four of the eyes were pseudophakic, and they had undergone prior vitrectomies. Three eyes had sclerally-fixated intraocular lenses, and one eye had a posterior chamber intraocular lens in the capsular bag, with a capsular tension ring due to partial zonular dehiscence. When comparing the vitrectomized eyes with reduced zonular/capsular bag complex integrity to the vitrectomized pseudophakic eyes with intact zonular/capsular bags, the former were significantly associated with an increased risk of anterior chamber implant migration (*P* = 0.008). The vitrectomized pseudophakic eyes, in contrast to the nonvitrectomized pseudophakic eyes, were significantly associated with an increased risk of anterior chamber implant migration (*P* = 0.009).

**Conclusions:**

The anterior chamber migration of an intravitreal dexamethasone implant is a serious complication. To minimize the risk of permanent corneal edema, immediate removal of the implant with a 20-gauge alligator forceps over a 2.75-mm long clear corneal tunnel is important. Those patients with insufficient zonular support, defects, or missing posterior capsular membranes and vitrectomy histories present a high risk of anterior chamber dexamethasone implant migration.

## Background

Intravitreal dexamethasone implants (Ozurdex®; Allergan, Irvine, CA, USA) have become efficacious treatment for macular edema associated with diabetic retinopathy, retinal vein occlusion, and noninfectious uveitis [1–4]. Ozurdex® is a sustained-release biodegradable implant that contains 0.7 mg of preservative-free dexamethasone. This rod-shaped implant, with its length of 6 mm and diameter of 0.46 mm, is inserted into the vitreous cavity using a 22-gauge needle. The implant releases the active ingredient within the vitreous chamber for up to 6 months after the intravitreal injection.

Although Ozurdex® can be an effective alternative to anti-vascular endothelial growth factor (anti-VEGF) treatment, it has a variety of ocular complications associated with its use [5]. In addition to the risk of cataract formation and steroid-induced glaucoma, migration of the dexamethasone implant into the anterior chamber is another possibly severe complication [6, 7]. The anterior chamber dislocation of a dexamethasone implant has recently been described in a few reports [8–12]. When migration into the anterior chamber occurs, the patient is at risk for corneal endothelial damage and corneal edema or decompensation. In those cases with permanent corneal decompensation, the patient must undergo a corneal transplantation [8, 12, 13]. Previous literature has suggested that the implant can maneuver through the pupil in aphakic eyes, through an iridectomy, and around an intraocular lens (IOL) to enter the anterior chamber [8, 10].

Based on the above information, the aims of our study were to report the incidence of and risk factors for the anterior chamber migration of dexamethasone implants, their management, and possible prevention. To the best of our knowledge, this is the first study at a university hospital in Europe to investigate the risk factors, management, and possible prevention of anterior chamber dexamethasone implant migration.

## Methods

For this study, we retrospectively reviewed 640 intravitreal dexamethasone implant injections (Ozurdex®) in 276 eyes of 234 patients from February 2011 through February 2018 at the University Eye Hospital in Tübingen, Germany. Each intravitreal dexamethasone implant was injected into the vitreous chamber in accordance with the manufacturer’s instructions under sterile conditions.

For all 640 eyes, preoperative and postoperative slit-lamp examinations were conducted, and best corrected visual acuity (BCVA), spectral domain optical coherence tomography (SD-OCT), and intraocular pressure (IOP) measurements were obtained. The ocular histories and surgical interventions prior to the dexamethasone injections were determined and documented, and those patients who experienced anterior chamber dexamethasone implant migration were identified. If possible, the reasons for the anterior chamber migrations were also identified.

This study was approved by the institutional review board of the University of Tübingen, and it adhered to the tenets of the Declaration of Helsinki.

### Statistical analyses

The statistical analysis of the data was managed using IBM SPSS Statistics for Windows, version 24.0 (IBM Corp., Armonk, NY, USA). The categorical data were analyzed using cross tabulations and Pearson’s chi-squared tests. Fisher’s exact tests were used as tests of association. The quantitative data were reported as the mean with the standard deviation. The odds ratios (ORs) were quoted with 95% confidence intervals (CIs), and a *p* < 0.05 was considered to be statistically significant.

## Results

From February 2011 to February 2018, a total of 640 consecutive intravitreal Ozurdex® implantations were performed in 234 patients at the University Eye Hospital in Tübingen. 136 of these patients were female and 98 male (female to male ratio = 58 to 42%), their mean age was 65 ± 13 years old (range = 23–88 years). The most common indications for the intravitreal dexamethasone implant injections were uveitis (316 injections in 107 patients), diabetic macular edema (113 injections in 33 patients), retinal vein occlusion (162 injections in 75 patients) and Irvine-Gass syndrome (49 injections in 19 patients).

Four eyes of four patients (0.63%) showed anterior chamber implant migrations, and all four of the eyes were pseudophakic, and they had undergone prior vitrectomies. Three eyes had sclerally-fixated IOLs, and one eye had a posterior chamber IOL in the capsular bag with a capsular tension ring due to partial zonular dehiscence. Two of these patients were treated because of persistent uveitis-related CME and the other two because of pseudophakic CME after complicated cataract surgery. The indication for treatment had no statistically significant influence on the anterior chamber migration rate of the dexamethasone implant (Table 1). However a trend was observed whereby patients treated because of Irvine-Gass syndrome (*P* = 0.068) have higher risk for Ozurdex® dislocation, but this trend did not meet our strict criteria for statistical significance (*P* < 0.05).

**Table 1:** Factors influencing the anterior chamber migration of an intravitreal dexamethasone implant. This table illustrates the significant influences of vitrectomized pseudophakic eyes in contrast to nonvitrectomized pseudophakic eyes and vitrectomized eyes with reduced zonular/capsular bag complex integrity in contrast to vitrectomized pseudophakic eyes with intact zonular/capsular bags on the risk of anterior chamber implant migration. Additionally this table illustrates the potential influence of treatment diagnosis for Ozurdex® dislocation

Factor	number	eyes in %	anterior chamber migration [%]	OR	95% CI	P
pseudophakic eyes with status post vitrectomy						
no	439	84.3	0	1.0	–	–
yes	82	15.7	4.9	50.4	2.7 945.2	0.009
vitrectomized eyes with reduced zonular/capsular bag complex						
no	68	82.9	0	1.0	–	–
yes	14	17.1	28.6	58.7	2.9 1171.2	0.008
Indications for intravitreal dexamethasone injections						
Retinal vein occlusion	162	25.3	0	1.0	–	–
Diabetes	113	17.7	0	1.4	0.03 72.69	0.9
Uveitis	316	49.4	0.006	2.6	0.12 54.13	0.5
Irvine-Gass syndrome	49	7.7	0.041	17.1	0.8 1362.5	0.068

% percentage, *OR* odds ratio, *CI* confidence interval

The anterior chamber implant migration incidence in the group with the vitrectomized eyes and pseudophakic intraoperative lenses was 4.9% (4 / 82). The group with the vitrectomized pseudophakic eyes, in contrast to the nonvitrectomized pseudophakic eyes, was significantly associated with an increased risk of anterior chamber implant migration (OR = 50.4, CI = 2.7–945.2, *P* = 0.009). The anterior chamber implant migration incidence in the group with the vitrectomized eyes and reduced zonular/capsular bag complex integrity was 28.6% (4 / 14). The vitrectomized eyes with the reduced zonular/capsular bag complex integrity, in contrast to the vitrectomized pseudophakic eyes with intact zonular/capsular bags, were significantly associated with an increased risk of anterior chamber implant migration (OR = 58.7, CI = 2.9–1171.2, *P* = 0.008) (Table 1).

The average time from the implantation to the anterior chamber migration detection was 27 days (range = 4–66 days), and all of the patients underwent surgical implant removals. The average time from the diagnosis to the implant explantation was 9 h (range = 2–18 h). All of the eyes had a corneal edema; two of them suffered from permanent edema that required a corneal transplantation.

Of the 640 eyes that underwent Ozurdex® implantations, 199 were phakic, 2 were aphakic, and 439 were pseudophakic. Two of the pseudophakic patients had iris-fixated posterior chamber intraocular lenses (PCIOLs), 6 of them had a sclerally-fixated PCIOLs, and 5 of them had sulcus-fixated PCIOLs with no posterior lens capsule (Table 2).

**Table 2:** The Ozurdex® implantation vitreous and lens statuses of 640 consecutive eyes

	Number of Eyes	Vitrectomized eyes (number of anterior chamber migration)
Pseudophakic	439	82 (4)
- Endocapsular	426	69 (1)
- Iris-fixated (Artisan)	2	2
- Scleral-fixated	6	6 (3)
- Sulcus-fixated	5	5
Phakic	199	1
Aphakic	2	2

### Patient 1

**Fig. 1:**
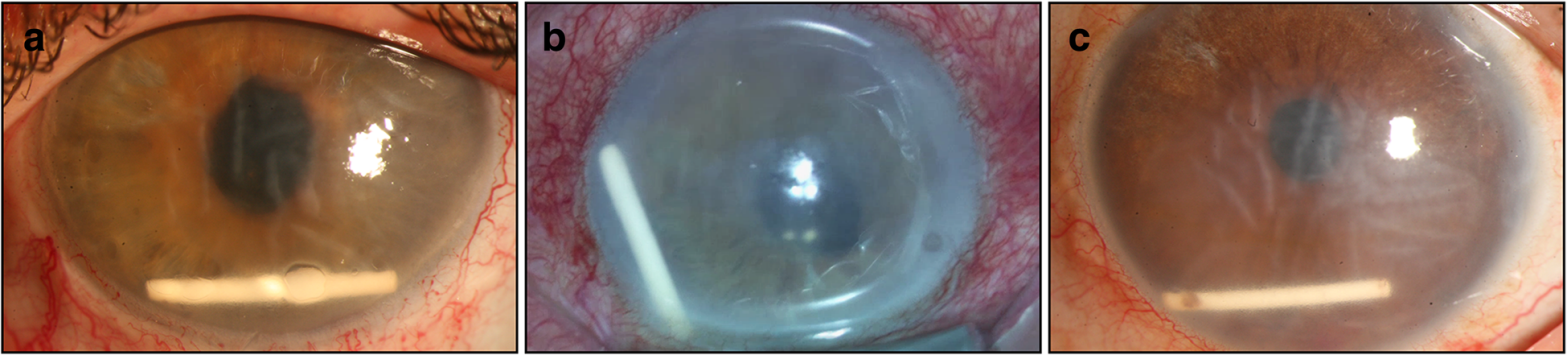
Slit-lamp photography showing the dexamethasone implant dislocated to the inferior angle of the anterior chamber, touching the corneal endothelium, in three different patients (**a–c**). Diffuse corneal edema and Descemet membrane folds can be seen

**Fig. 2:**
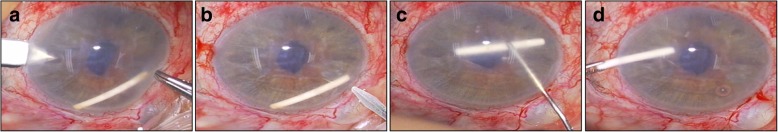
Surgical technique for the explantation of a dislocated Ozurdex® implant. The surgical technique involved creating a temporally located clear corneal tunnel with a 2.75-mm slit knife (**a**) and paracentesis at the 10 o’clock position (**b**). Viscoelastic material was injected through the paracentesis into the anterior chamber, and the Ozurdex® implant was freed from the anterior chamber angle (**c**). Twenty-gauge alligator forceps were used to grip the implant at its long axis in order to avoid disintegration of the brittle implant (**d**)

A 47-year-old woman with noninfectious chronic uveitis and persistent cystoid macular edema (CME) was referred to our institution due to a dislocated IOL in the vitreous chamber in August 2010. A vitrectomy was performed, and the dislocated IOL was removed and exchanged with a sclerally-fixated IOL using a knotless zigzag-shaped intrascleral suture (Z-suture) [14]. Due to the persistent uveitis-related CME, Ozurdex® was injected into the left eye. At that moment, the BCVA in her left eye was 20/100. Thirteen days after the Ozurdex® implantation, the patient presented with diffuse corneal edema, and her visual acuity was counting fingers. The Ozurdex® implant was detected in the inferior angle of the anterior chamber (Fig. 1a). Eighteen hours after detection, the implant was removed. This surgical technique involved a temporally located clear corneal tunnel created with a 2.75-mm slit knife (Fig. 2a) and paracentesis at the 10 o’clock position (Fig. 2b). Viscoelastic material was injected through the paracentesis into the anterior chamber, and the Ozurdex® implant was freed from the anterior chamber angle (Fig. 2c). Twenty-gauge alligator forceps were used to grip the implant at its long axis in order to avoid the disintegration of this brittle implant (Fig. 2d).

Following surgery, the BCVA in her left eye was 20/200. An anterior segment examination showed diffuse corneal edema and a stable, well-positioned, sclerally-fixated IOL (Fig. 1b). Six months later, a Descemet membrane endothelial keratoplasty (DMEK) procedure was performed. Two and 9 days after the DMEK, rebubbling procedures were performed using an intracameral air injection due to a partially detached graft. Three months later, her BCVA had improved to 20/100.

### Patient 2

In 2011, a 76-year-old man was referred to our institution with a dislocated pseudophakic IOL due to pseudoexfoliation syndrome (PEX). Five years earlier, he underwent a unilateral cataract extraction with a capsular tension ring and an endocapsular IOL implantation. After performing an explantation of the capsular bag, capsular tension ring, and dislocated IOL, a limited anterior vitrectomy and implantation of a sclerally-fixated IOL were performed [14]. This patient developed pseudophakic CME due to Irvine-Gass syndrome. Because the CME did not respond to topical steroids, nonsteroidal anti-inflammatory agents (NSAIDs), peribulbar steroid injections, or anti-VEGF agents, an uncomplicated Ozurdex® injection was performed. At that time, his BCVA was 20/50.

Twenty-four days after the fourth Ozurdex® injection, the patient was referred with complaints of worsening blurry vision and discomfort in his left eye due to corneal decompensation after the migration of the dexamethasone implant into the anterior chamber (Fig. 1b). His BCVA had decreased to 20/100. A clinical diagnosis of corneal decompensation with bullous keratopathy was made, and an Ozurdex® explantation was proposed and agreed upon. Two hours after detection, he underwent the explantation procedure using the same technique as that described in the previous case. Three months after the dexamethasone implant explantation, the corneal edema had decreased, and his visual acuity was 20/50.

### Patient 3

An 84-year-old woman underwent a complicated cataract surgery with a vitrectomy and a sclerally-fixated IOL in March 2017 [14]. She developed pseudophakic CME due to Irvine-Gass syndrome. At that time, her BCVA was 20/50. Due to her poor response to topical NSAIDS, oral carbonic anhydrase inhibitors, and a peribulbar steroid injection, an Ozurdex® injection was performed. Sixty-six days after insertion, the intravitreal Ozurdex® implant had migrated into the anterior chamber. In February 2018, this patient’s anterior segment examination showed diffuse corneal edema and an Ozurdex® implant in the inferior angle of the anterior chamber. At that time, her visual acuity had decreased to hand movement. Three hours after detection, the Ozurdex® implant was removed from the anterior chamber; however, her vision remained at hand movement with bullous keratopathy. This patient is being scheduled for a DMEK.

### Patient 4

A 69-year-old woman suffering from noninfectious chronic uveitis with persistent CME in her left eye was referred to our institution. In November 2006, a cataract surgery was performed with a capsular tension ring implantation into the capsular bag due to partial zonular dehiscence. In order to exclude infectious uveitis, a diagnostic vitrectomy and surgical posterior capsulotomy were performed. After this surgery, the BCVA in her left eye was 20/100. Due to the persistent uveitis-related CME, an Ozurdex® injection was administered in the left eye. This patient returned 4 days later with diffuse corneal edema and Descemet membrane folds (Fig. 1c). An anterior segment examination showed anterior chamber dislocation of the dexamethasone implant. At this point, her visual acuity was 20/400. Due to the corneal decompensation and decrease in vision, the dexamethasone implant in the anterior chamber was removed surgically. Postoperatively, her BCVA had improved to 20/100, and her cornea was clear.

## Discussion

Anterior chamber intravitreal dexamethasone implant migrations have been documented previously. For example, Pardo-López et al. first described an Ozurdex® migration into the anterior chamber in a postvitrectomy eye with an iris-claw IOL [12]. In addition, different risk factors for anterior chamber migration have described in a few reports. Some studies have reported that, in aphakic vitrectomized eyes, the implant can maneuver through a pupil, through an iridectomy, and around an IOL to enter the anterior chamber [8, 10, 15, 16]. All of those cases had common histories of a vitrectomy and either weak zonules, a defect, or a missing posterior capsular membrane. Our study confirmed these risk factors for anterior chamber migration. All four of the eyes in our study underwent prior vitrectomies; three of them had no posterior capsular membrane, and one had partial zonular dehiscence.

An anterior chamber implant migration in a vitrectomized eye can be facilitated by the lack of an anterior hyaloid membrane. The lack of this membrane creates a communication between the vitreous cavity and the anterior chamber. This communication also exists in partially anterior vitrectomized eyes and, in combination with a missing capsular bag (Patient 2), can lead to an anterior chamber migration. The vitreous fluid can “hold” the implant; however, after a vitrectomy, the vitreous cavity is filled with aqueous fluid, which can allow implant movement [15]. For this reason, vitrectomized eyes are at a higher risk for Ozurdex® movement than nonvitrectomized eyes. Additionally, reduced integrity of the zonular/capsular bag complex can cause it to dislocate into the anterior chamber. In our study, the vitrectomized eyes with reduced zonular/capsular bag complex integrity, in contrast to the vitrectomized pseudophakic eyes with intact zonular/capsular bags, were significantly associated with an increased risk of anterior chamber implant migration. Additionally, the vitrectomized pseudophakic eyes, in contrast to the nonvitrectomized pseudophakic eyes, were significantly associated with an increased risk of anterior chamber implant migration.

Two of the patients in our series were aphakic and vitrectomized. In these two patients we did not realize anterior chamber dexamethasone implant migration or corneal edema. Especially, aphakic–vitrectomized eyes have a high risk of anterior chamber migration [8]. The vitreous cavity is filled with aqueous fluid and therefore the implant has the potential to migrate forth and back with minimal resistance and changing postures. For this reason there are two possibilities in our two aphakic patients. On the one hand there was no anterior chamber migration or on the other hand the implant migrated forth and back without symptoms. Accordingly, use of dexamethasone intravitreal implant should be avoided in aphakic eyes with rupture of the posterior lens capsule.

A trend was observed whereby patients treated because of Irvine-Gass syndrome have higher risk for Ozurdex® dislocation, but this trend did not meet our strict criteria for statistical significance. One reason for this trend could be that the risk for postoperative CME is significant higher in eyes with complicated cataract surgery (with posterior capsular tear) than in uneventful surgeries [17].

Implant migration into the anterior chamber is a serious adverse event. The anterior chamber migration of a dexamethasone intravitreal implant may cause corneal edema and permanent endothelial decompensation due to its direct contact with the endothelium, as well as mechanical trauma and/or chemical toxicity [18]. Therefore, the surgical removal of the implant must be performed as soon as possible. In previous reports, different strategies to manage the dislocated implant have been reported. For example, Kishore et al. described a noninvasive intervention to reposition the Ozurdex® implant back into the vitreous cavity using supine positioning after dilating the pupil [19]. Another minimally invasive surgical technique included the repositioning of the implant in the posterior chamber with a needle under topical anesthesia [11, 16]. These techniques can be used in those cases with no corneal decompensation. One potential risk factor for these techniques is that the implant can re-migrate into the anterior chamber. For this reason, these patients must avoid prone positioning, and they must use pilocarpine drops to reduce the pupil size.

The majority of the cases reported in the literature have required implant explantations to avoid corneal damage and permanent corneal edema. In our study, diffuse corneal edema and Descemet membrane folds occurred in all four of the patients with Ozurdex® migrations. Moreover, two out of the four patients required corneal transplantations. Especially, eyes with histories of multiple previous surgeries exhibit a higher risk for irreversible corneal edema due to a reduced endothelial cell count. If a patient presents with an implant dislocated into the anterior chamber, immediate removal or repositioning must be done in order to avoid permanent damage to the corneal endothelium.

In order to avoid anterior chamber implant migration, Mateo et al. described an intravitreal scleral fixation of Ozurdex® using 10–0 nonabsorbable polypropylene suturing to the pars plana [20].

## Conclusion

Ophthalmologists should be aware of anterior chamber implant migration, which is a potentially serious adverse event that can occur after a vitrectomy. In order to minimize the risk of permanent corneal edema, immediate removal of the implant is important. Those patients with insufficient zonular support, defects, or a missing posterior capsular membrane and a vitrectomy history present a high risk of anterior chamber dexamethasone implant migration. Caution is recommended in these patients, and alternative treatments, such as the intravitreal application of triamcinolone, anti-VEGF agents, or an intravitreal scleral fixation of the implant, may be considered.

## Abbreviations

Anti-VEGF: Anti-vascular endothelial growth factor
BCVA: Best corrected visual acuity
CI: Confidence interval
CME: Cystoid macular edema
DMEK: Descemet membrane endothelial keratoplasty
IOL: Intraocular lens
IOP: Intraocular pressure
NSAID: Nonsteroidal anti-inflammatory agent
OR: Odds ratio
PCIOL: Posterior chamber intraocular lens
PEX: Pseudoexfoliation syndrome
SD-OCT: Spectral domain optical coherence tomography

## References

[1] Haller JA, Bandello F, Belfort R, Blumenkranz MS, Gillies M, Heier J, Loewenstein A, Yoon YH, Jiao J, Li XY, Whitcup SM, Li J, Ozurdex GENEVA Study Group (2011). Dexamethasone intravitreal implant in patients with macular edema related to branch or central retinal vein occlusion twelve-month study results. Ophthalmology.

[2] Haller JA, Bandello F, Belfort R, Blumenkranz MS, Gillies M, Heier J, Loewenstein A, Yoon YH, Jacques ML, Jiao J, Li XY, Whitcup SM, OZURDEX GENEVA Study Group (2010). Randomized, sham-controlled trial of dexamethasone intravitreal implant in patients with macular edema due to retinal vein occlusion. Ophthalmology.

[3] Lowder C, Belfort R, Lightman S, Foster CS, Robinson MR, Schiffman RM, Li XY, Cui H, Whitcup SM (2011). Ozurdex HURON study group. Dexamethasone intravitreal implant for noninfectious intermediate or posterior uveitis. Arch Ophthalmol.

[4] Boyer DS, Yoon YH, Belfort R, Bandello F, Maturi RK, Augustin AJ, Li XY, Cui H, Hashad Y, Whitcup SM, Ozurdex MEAD Study Group (2014). Three-year, randomized, sham-controlled trial of dexamethasone intravitreal implant in patients with diabetic macular edema. Ophthalmology.

[5] Mayer WJ, Remy M, Wolf A, Kook D, Kampik A, Ulbig M, Reznicek L, Haritoglou C (2012). Comparison of intravitreal bevacizumab upload followed by a dexamethasone implant versus dexamethasone implant monotherapy for retinal vein occlusion with macular edema. Ophthalmologica..

[6] Pielen A, Feltgen N, Isserstedt C, Callizo J, Junker B, Schmucker C (2013). Efficacy and safety of intravitreal therapy in macular edema due to branch and central retinal vein occlusion: a systematic review. PLoS One.

[7] Meyer LM, Schönfeld CL (2013). Secondary glaucoma after intravitreal dexamethasone 0.7 mg implant in patients with retinal vein occlusion: a one-year follow-up. J Ocul Pharmacol Ther.

[8] Khurana RN, Appa SN, McCannel CA, Elman MJ, Wittenberg SE, Parks DJ, Ahmad S, Yeh S (2014). Dexamethasone implant anterior chamber migration: risk factors, complications, and management strategies. Ophthalmology.

[9] Madi HA, Morgan SJ, Ghosh S (2017). Corneal graft failure due to migration of Ozurdex™ implant into the anterior chamber. Am J Ophthalmol Case Rep.

[10] Kang H, Lee MW, Byeon SH, Koh HJ, Lee SC, Kim M (2017). The clinical outcomes of surgical management of anterior chamber migration of a dexamethasone implant (Ozurdex®). Graefes Arch Clin Exp Ophthalmol.

[11] Pacella F, Agostinelli E, Carlesimo SC, Nebbioso M, Secondi R, Forastiere M, Pacella E (2016). Management of anterior chamber dislocation of a dexamethasone intravitreal implant: a case report. J Med Case Rep.

[12] Pardo-López D, Francés-Muñoz E, Gallego-Pinazo R, Díaz-Llopis M (2012). Anterior chamber migration of dexametasone intravitreal implant (Ozurdex®). Graefes Arch Clin Exp Ophthalmol.

[13] Bansal R, Bansal P, Kulkarni P, Gupta V, Sharma A, Gupta A (2012). Wandering Ozurdex(®) implant. J Ophthalmic Inflamm Infect.

[14] Szurman P, Petermeier K, Aisenbrey S, Spitzer MS, Jaissle GB (2010). Z-suture: a new knotless technique for transscleral suture fixation of intraocular implants. Br J Ophthalmo.

[15] Stepanov A, Codenotti M, Ramoni A, Prati M, Jiraskova N, Rozsival P, Bandello F (2016). Anterior chamber migration of dexamethasone intravitreal implant (Ozurdex®) through basal iridectomy (Ando) in a pseudophakic patient. Eur Joul Ophthalmol.

[16] Vela JI, Crespí J, Andreu D (2012). Repostioning of dexamethasone implant (Ozurdex) migrated into the anterior chamber. Int. Ophthalmol..

[17] Akçay Bİ, Bozkurt TK, Güney E, Unlü C, Erdogan G, Akcali G, Bayramlar H (2012). Quantitative analysis of macular thickness following uneventful and complicated cataract surgery. Clin Ophthalmol.

[18] Chen WL, Lin CT, Yao CC, Huang YH, Chou YB, Yin HS, Hu FR (2006). In-vitro effects of dexamethasone on cellular proliferation, apoptosis, and Na+−K+-ATPase activity of bovine corneal endothelial cells. Ocul Immunol Inflamm.

[19] Kishore SA, Schaal S (2013). Management of anterior chamber dislocation of dexamethasone implant. Ocul Immunol Inflamm.

[20] Mateo C, Alkabes M (2014). Scleral fixation of dexamethasone intravitreal implant (OZURDEX®) in a case of angle-supported lens implantation. Int Opthalmol.

